# Age-Specific Effects of Mirror-Muscle Activity on Cross-Limb Adaptations Under Mirror and Non-Mirror Visual Feedback Conditions

**DOI:** 10.3389/fnagi.2015.00222

**Published:** 2015-12-01

**Authors:** Paola Reissig, Tino Stöckel, Michael I. Garry, Jeffery J. Summers, Mark R. Hinder

**Affiliations:** ^1^Human Motor Control Laboratory, School of Medicine, Faculty of Health, University of TasmaniaHobart, TAS, Australia; ^2^Faculty of Health Graduate Research Program, University of TasmaniaHobart, TAS, Australia; ^3^Sport and Exercise Psychology Unit, Department of Sport Science, University of RostockRostock, Germany; ^4^School of Sport and Exercise Sciences, Faculty of Science, Liverpool John Moores UniversityUK

**Keywords:** cross-limb transfer, ageing, mirror therapy, transcranial magnetic stimulation, unilateral ballistic movement task

## Abstract

Cross-limb transfer (CLT) describes the observation of bilateral performance gains due to unilateral motor practice. Previous research has suggested that CLT may be reduced, or absent, in older adults, possibly due to age-related structural and functional brain changes. Based on research showing increases in CLT due to the provision of mirror visual feedback (MVF) during task execution in young adults, our study aimed to investigate whether MVF can facilitate CLT in older adults, who are known to be more reliant on visual feedback for accurate motor performance. Participants (*N* = 53) engaged in a short-term training regime (300 movements) involving a ballistic finger task using their dominant hand, while being provided with either visual feedback of their active limb, or a mirror reflection of their active limb (superimposed over the quiescent limb). Performance in both limbs was examined before, during and following the unilateral training. Furthermore, we measured corticospinal excitability (using TMS) at these time points, and assessed muscle activity bilaterally during the task via EMG; these parameters were used to investigate the mechanisms mediating and predicting CLT. Training resulted in significant bilateral performance gains that did not differ as a result of age or visual feedback (both *p* > 0.1). Training also elicited bilateral increases in corticospinal excitability (*p* < 0.05). For younger adults, CLT was significantly predicted by performance gains in the trained hand (*β* = 0.47), whereas for older adults it was significantly predicted by mirror activity in the untrained hand during training (*β* = 0.60). The present study suggests that older adults are capable of exhibiting CLT to a similar degree to younger adults. The prominent role of mirror activity in the untrained hand for CLT in older adults indicates that bilateral cortical activity during unilateral motor tasks is a compensatory mechanism. In this particular task, MVF did not facilitate the extent of CLT.

## Introduction

Unilateral training can induce performance increases in both the trained and untrained limb. Such bilateral performance gains are known as cross-limb transfer (CLT) or cross-education and have been shown in a variety of strength and skill acquisition tasks (for an overview, see Carroll et al., 2006; Farthing, 2009; Ruddy and Carson, 2013). Recent work has suggested that ageing may be associated with a reduction in the extent to which CLT is manifested relative to that observed in younger adults. However, the exact mechanisms underlying such an effect are not yet completely understood.

Ageing is known to be associated with changes in motor performance (for an overview, see Seidler et al., 2010), with increased bilateral activation (at the cortical or muscle level) during unilateral training observed across a number of tasks (Mattay et al., 2002; Ward and Frackowiak, 2003; Hinder et al., 2011). Such increases in mirror muscle activity in older adults are suggested to be caused by changes in the neural control mechanisms underpinning movement performance (Fujiyama et al., 2009; Hinder et al., 2011, 2012), such as a decreased ability to modulate intra- and interhemispheric inhibitory mechanisms (for a review, see Hoy et al., 2004; Talelli et al., 2008). As increased bilateral activation (at the cortical or muscle level) has been shown to be associated with enhanced motor performance in older adults (Mattay et al., 2002; Bodwell et al., 2003; Naccarato et al., 2006; Hinder et al., 2011) it was previously hypothesized that greater mirror activity (i.e., greater bilateral cortical activity measured via TMS) may promote greater transfer in older adults. However, despite an increased level of mirror activity in the older adults, Hinder et al. (2011) did not find a correlation between mirror activity and transfer and thus suggested the inability to regulate mirror activity may actually limit the transfer of motor skills in the advanced age.

As ageing is associated with an increased reliance on visual control and older adults benefit from visual feedback for accurate motor performance (Swinnen et al., 1998; Voelcker-Rehage, 2008) we propose that another potential reason for the observed absence/decrease of transfer in previous studies (Hinder et al., 2011; Parikh and Cole, 2013) could have been the absence of a specific focus of attention on available visual feedback. Specifically, neither Hinder et al. (2011) nor Parikh and Cole (2013), who studied CLT in a group of younger and older people using the same motor task, instructed their participants to maintain a focus of attention on visual feedback *during* task execution.

A special type of visual feedback is mirror-visual feedback (MVF), whereby a mirror image of one (usually active) limb is superimposed over the actual position of the other (usually inactive) limb by means of a mirror placed in a person’s midsagittal plane. Mirror training (using MVF) was introduced by Ramachandran and Rogers-Ramachandran (1996) as a psychophysiological technique to alleviate phantom-limb pain. Although the exact underlying neural mechanisms of this phenomenon are incompletely understood (Garry et al., 2005; Ramachandran and Altschuler, 2009; Carson and Ruddy, 2012; Reissig et al., 2014), behavioral evidence indicates beneficial effects of mirror therapy within stroke rehabilitation (Altschuler et al., 1999; Yavuzer et al., 2008) or the treatment of chronic regional pain syndrome (McCabe et al., 2003). MVF could be viewed as a form of augmented visual feedback (Howatson et al., 2013), which has been shown to increase motor learning (Schmidt and Lee, 2011). More recently, MVF has also been demonstrated to be advantageous compared to more standard visual feedback (i.e., “normal” vision of the hand undertaking the task) when applied in a unilateral motor task, leading to enhanced CLT in younger people (Lappchen et al., 2012; Nojima et al., 2012). An approach to increase bilateral behavioral benefits via unilateral training appears particularly useful during rehabilitation of a limb following stroke or traumatic injury. This is particularly the case for older adults, as even short periods of immobilization (e.g., splinting or casting of a limb due to a fall-related injury) have been shown to result in a significant loss of strength and consequently affect older adults’ functional ability to maintain an independent lifestyle. Intervention programmes should aim at minimizing the loss of strength during periods of immobilization, and ensure a quicker return to independent living. The utilization of *unilateral* training paradigms that would result in bilateral performance changes are thus very appealing in an ageing population as they could be used during the period in which the affected limb is too weak to undertake physical training itself (or is indeed immobilized/cast due to fracture); as such this technique may maintain functional capacity by reducing the extent of functional losses during periods of immobility or weakness. The current study therefore aimed to determine whether CLT may be enhanced by augmented sensory feedback (i.e., MVF) in older adults, who may have underlying deficits in the ability to exhibit CLT (Hinder et al., 2011; Parikh and Cole, 2013).

For the current experiment we employed a well-studied goal-directed ballistic finger movement task (i.e., aim to achieve peak acceleration; Carroll et al., 2008; Lee et al., 2010; Hinder et al., 2011) that is known to share neural mechanisms with strength training paradigms (Selvanayagam et al., 2011) and moreover has been shown to elicit a strong neural drive emerging from the contralateral primary motor cortex (M1). Activation of the motor cortex due to voluntary movements has previously been shown to facilitate cortical activation of the ipsilateral cortical areas, with an increasing force leading to increased bilateral activation (Dettmers et al., 1996; Muellbacher et al., 2000; for an overview, see Carroll et al., 2006). Assuming CLT occurs substantially due to neural mechanisms at the level of the cortex (Carroll et al., 2006; Lee et al., 2010) an activation of interhemispheric connections between left M1 – right M1 might be a crucial prerequisite for CLT to occur. We therefore propose that a combination of a goal-directed motor task that strongly engages the contralateral M1 combined together with MVF may lead to greater behavioral benefit (i.e., bilateral performance increase) in older adults when compared to younger adults.

We were also interested in investigating whether certain (behavioral/neurophysiological) parameters measured during the unilateral training period could explain performance increases observed in the untrained hand for both younger and older adults. Specifically, we were interested in the influence of two particular variables: firstly, whether the extent of bilateral muscle activation exhibited during the acquisition of the present ballistic unilateral motor learning task was associated with the amount of subsequent transfer. This proposition was based on a previous study (Graziadio et al., 2015) showing greater transfer in older (compared to younger) adults in the feedforward control component of a motor learning task (previously associated with bilateral cortical activation only in older adults). In contrast, transfer was reduced in older adults relative to the younger adults in the feedback control component of the same task (previously associated with bilateral activation in both younger and older adults). Because our ballistic acceleration task is driven by feedforward mechanisms (and thus associated with predominantly unilateral cortical activity in younger adults), we hypothesized that any bilateral activity in older adults may also facilitate CLT. Secondly, we aimed to determine the extent to which the degree of performance improvements in the trained hand is associated with the subsequent degree of CLT in the untrained hand. Considering age-related changes with regard to behavioral and neural control of movements (Fujiyama et al., 2009; Hinder et al., 2011, 2012), and interpreting previous findings of reduced CLT in older adults despite comparable improvements in the trained limb (Hinder et al., 2011; Parikh and Cole, 2013) as possible evidence for a change in the mechanisms underlying CLT with advancing age, we were interested in investigating whether there was a difference across age in the underlying factors predicting successful CLT.

## Materials and Methods

### Participants

Twenty-seven younger (mean *age* = 26.1 years, *SD* = 5.3, 9 men) and 26 older (mean *age* = 69.6 years, *SD* = 5.6, 12 men) adults participated in the experiment. Fifty-one declared themselves as right-handed, two as left-handed. All participants had normal or corrected-to-normal vision and were screened for contraindications to transcranial magnetic stimulation (TMS). Additionally, a medical history questionnaire revealed that they were free from any known neuromuscular disorders and did not have a history of neurological illnesses that might affect neurophysiological measures (as assessed by TMS). Finally, all participants were community dwelling with no known cognitive deficits. The experimental procedures were approved by, and carried out in accordance with local ethical guidelines laid down by the Tasmanian Human Research Ethics Committee Network, and conformed to the declaration of Helsinki. Prior to the beginning of the experiment participants asked any questions regarding techniques and procedures and when they were satisfied, signed an informed consent form. Participants either received course credit, or were reimbursed $20.

### Movement Task

Participants were seated in a height adjustable chair with their forearms pronated and hands resting on a horizontal board to standardize hand position and isolate movements to their index finger. Participants were asked to perform unilateral ballistic abduction movements with their left and right index finger (see Hinder et al., 2013), while keeping the rest of the hand still. The aim of the task was to maximize the horizontal peak acceleration of each movement, measured using an accelerometer (Dytran Insturments, Chatsworth, CA, USA/Endevco Corp. San Juan Capistrano, CA, USA) attached to the index finger with a plastic splint and tape.

### Experimental Design and Procedure

**Figure 1:**
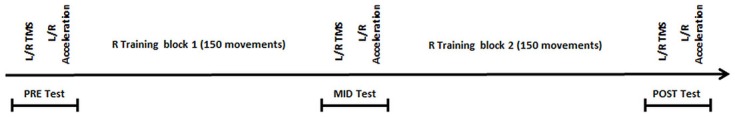
**Experimental timeline.** L = left; R = right.

**Figure 2:**
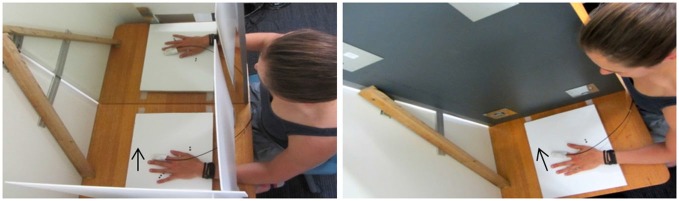
**Visual feedback conditions: Mirror Vision (left) and Active Vision (right)**.

Prior to motor training (pre-test), we measured corticospinal excitability and intracortical inhibition in both hemispheres. The neurophysiological testing was followed by a bilateral assessment of participants’ motor performance (behavioral testing), consisting of 10 trials of the ballistic finger movement performed at 0.5 Hz paced by an auditory metronome. Subsequently, participants performed two blocks of 150 trials of the same task with their dominant hand and were provided with one of two forms of feedback during performance. Participants in the Active Vision (AV) group (younger group: 12, older group: 14) were asked to focus on their active hand, while vision of their inactive hand was occluded with a wooden box. For the Mirror Vision (MV) group (younger group: 15, older group: 12), a mirror was placed vertically in the midsagittal plane and participants saw a mirror reflection of their active hand. Direct vision of the inactive hand was not possible due to the positioning of the mirror; however, the mirror image of the active hand appeared superimposed on top of the obscured inactive hand. A custom-built stand, situated between participants’ upper body and their active hand, also prevented direct vision of the active hand (Figure 1). Auditory feedback in the form of a high or a low pitch tone was provided after each trial, informing participants whether peak acceleration on the preceding trial had been better (high tone) or worse (low tone) than the previous trial. Participants were familiarized with both tones before the start of the experiment to ensure their ability to distinguish them. The experimenter encouraged participants on a regular basis and reminded them to “move as fast as possible” and to “produce/achieve as many high tones as possible”. A rest period of 30 s was given every 15 trials, therefore dividing the training period into 20 sub-blocks. We collected participants’ neurophysiological and behavioral data bilaterally after each training block (i.e., mid-test and post-test respectively) in a counterbalanced order (right/left hemisphere and right/left hand), but with the neurophysiological testing always preceding behavioral testing. Figure 2 outlines the experimental procedure.

### Electromyographic Recordings

Bilateral electromyographic (EMG) recordings were obtained from the left and right first dorsal interosseus (FDI) muscles, the muscle primarily responsible for the finger abduction task. Participants’ skin was prepared with a lightly abrasive gel and cleaned with an alcohol wipe before attaching Ag/AgCl electrodes (Meditrace 130, Tyco Healthcare, Mansfield, MA, USA) in a belly-tendon montage. EMG signals were amplified (X1000) and a notch filter (50 Hz) was applied prior to sampling using a 16-bit AD system (Power 1401, CED Limited, Cambridge, UK). Collected data was stored on a computer for subsequent offline analysis.

### Transcranial Magnetic Stimulation

TMS was used to investigate corticospinal excitability and short-interval intracortical inhibition (SICI) of the motor pathways from the left and right motor cortices (*l*M1 and *r*M1) at three different time points (i.e., before, between and after the two training blocks). TMS was delivered by two Magstim 200 magnetic stimulators (Magstim Company, UK) connected by a Bistim unit and a figure-of-eight coil (70 mm diameter). The optimal positions on the motor cortex (i.e., motor hotspots) at which a suprathreshold stimulation consistently elicited the largest motor evoked potentials (MEPs) in the left and the right FDI were determined and marked on the scalp. Resting motor thresholds (RMT), defined as the lowest TMS intensity needed to elicit at least three out of five MEPs ≥ 50 μV (Carroll et al., 2001; Reissig et al., 2014), were then determined for both target muscles using a posterior-to-anterior coil positioning (i.e., coil at ~45° to the midline and in a plane tangential to the scalp surface leading to a posterior-to-anterior-induced current in the cortex).

During all three TMS sessions we administered 20 alternating single-pulses and paired-pulses to the motor cortices of the left and right hemisphere. Ten single-pulses were applied to assess corticospinal excitability using a suprathreshold stimulation intensity (130% RMT), and ten paired-pulses were applied to assess intracortical inhibitory processes. SICI was measured by applying a subthreshold conditioning stimulus before a suprathreshold test stimulus (130% RMT) with an interstimulus interval (ISI) of 3 ms (Kujirai et al., 1993). Following Garry and Thomson (2009) a fixed test (130% RMT) and fixed conditioning stimulus intensity (70% RMT) was employed to measure SICI. The ratio of the paired-pulse to single-pulse MEP amplitudes was used as an indication level of SICI.

### Data Acquisition and Analysis

Custom-written CED (Cambridge, UK) Signal programs were used to sample kinematic and EMG data of each finger movement at 2 kHz for a duration of 1500 ms starting at 500 ms before the “go” tone. Acceleration data were low-pass filtered at 20 Hz prior to analysis, and peak acceleration was defined as the first peak in the horizontal acceleration.

Raw horizontal peak acceleration values were determined for both the left and the right hand at pre-, mid-, and post-test (*ACC*) and averaged across the ten trials. Peak acceleration obtained at mid- and post-test was normalized (*nACC*) to those values obtained at pre-test for each hand (i.e., trained hand acceleration was normalized to *ACC*_trained_ in the pre-test, untrained hand acceleration was normalized to *ACC*_untrained_ in the pre-test). A value of one was subsequently subtracted from these normalized accelerations to yield normalized change (*ΔACC*_untrained_ *ΔACC*_untrained_).

Peak acceleration, obtained during the two training blocks, was calculated in a similar way. For each of the 20 sub-blocks (see *experimental design*) we calculated an average raw peak acceleration value (*ACCtraining*) obtained during training. The average raw peak acceleration value (i.e., *ACCtraining—*see “Materials and Methods” Section) from the penultimate block (training period 2: movements 270–285) was then normalized to the average raw peak acceleration value from the first block (training period 1: movement 1–15) to obtain a variable (*nACCtraining*) describing performance gains in the trained hand over the duration of training using a single variable.

Responses to TMS at all three test points were excluded from further analysis if root-mean-squared EMG values exceeded 0.025 mV in the period 115–15 ms prior to each TMS pulse. In the remaining trials, the peak-to-peak MEP amplitudes elicited in the FDI contralateral to the stimulated hemisphere were calculated in the 50 ms window commencing 15 ms after TMS delivery. Single-pulse MEP amplitudes (*MEP*) were averaged and normalized to the MEPs obtained during the pre-test in each hand on a participant-by-participant basis (*nMEP*). Paired-pulse MEP amplitudes in both hands were determined for each trial in the pre-, mid-, and post-test and divided by the corresponding* MEP* of the same test-block to calculate a SICI ratio for each test-block (*SICI*). Accordingly, SICI < 1 indicates inhibition is present, with lower SICI indicating greater inhibition. The same procedure as described above for MEPs was then applied to calculate normalized SICI (*nSICI*).

EMG data of the two training blocks were rectified and low-pass filtered (20 Hz) and subsequently analyzed to quantify the movement-related muscle activity (trained hand) and corresponding mirror activity (untrained hand) prior to and during training. The peak EMG amplitude of the trained hand was determined and movement onset and offset were defined as the time at which EMG activity first increased above 4× the background EMG determined before movement onset and the time at which muscle activity of the active FDI first dropped below 0.2× the peak amplitude respectively (Carroll et al., 2008; Hinder et al., 2011). The average EMG activity of the trained (active) hand (*EMGtraining_trained_*) was then calculated for this time-period, minus the background EMG exhibited prior to movement onset for each trial in the pre-test and in both training blocks respectively. The average EMG activity of the untrained (inactive) hand (*EMGtraining*_untrained_) was established for the same time period using movement onset and offset as calculated above. For the training trials only, we then normalized the mirror activity in the untrained hand (as calculated above) to the EMG in the trained hand (for the same time period) for each trial. This method allowed us to refer to EMG activity in the inactive hand expressed as a percentage of the EMG activity in the active hand. We then averaged across all training trials to yield one value that represented the extent of mirror activation during training (*EMGtraining*_untrained_).

### Statistical Analysis

We separately analyzed our test- and training-related dependent variables relating to task performance (*ACC, nACC, nACCtraining*), cortical excitability (*MEP, nMEP*) and inhibition SICI (*SICI, nSICI*), and volitional muscle activity during the motor task (*EMGtraining*_untrained_) in multiple steps using various mixed model and between subject analyses of variance.

Specifically, 2 (hand: left, right) × 2 (feedback: Mirror Vision, Active Vision) × 2 (age: younger, older) analyses of variances (Mixed ANOVAs) were initially employed in order to check for differences at pre-test for *ACC*, *MEP* and* SICI*. Next, in order to investigate test-related behavioral and neurophysiological changes in the trained and the untrained hand and hemisphere (relative to the pre-test), we subsequently examined *nACC*, *nMEP* and* nSICI* using 2 (time: mid, post) × 2 (hand: left, right) × 2 (feedback: Mirror Vision, Active Vision) × 2 (age: younger, older) analyses of variance (Mixed ANOVAs) for each dependent variable separately. In addition, we interpreted training-induced changes from pre-test to mid-test based on confidence interval assessment.

In a next step, we investigated changes in task performance in the trained hand as well as differences in the average level of EMG (mirror) activity in the untrained hand during training performing separate 2 (feedback: Mirror Vision/Active Vision) × 2 (age: younger, older) between-subject analyses of variance using *nACCtraining* and *EMGtraining*_untrained_.

Separate multiple regression analyses for each age group were employed to identify main predictors of *ΔACC_untrained_*, and to assess a possible relationship between *ΔACC*_untrained_ and training-related variables *nACCtraining* and *EMGtraining*_untrained_. Furthermore, we were also interested in possible relationships between *ΔACC*_untrained_ and the test-related variables *ΔACC*_untrained_, *nMEP*_trained_, *nMEP*_untrained_, *nSICI*_trained_, and *nSICI*_untrained_ which represent changes in test performance and neural excitability/inhibition that occurred as a function of training. The two training-related variables (*nACCtraining* and *EMGtraining*_untrained_) were entered into the regression analysis (Enter Method) as a first cluster of potential predictors of *ΔACC*_untrained_, and subsequently complemented by a second cluster of predictors using the test-related variables (*ΔACC*_untrained_, *nMEP*_trained_, *nMEP*_untrained_, *nSICI*_trained_, and *nSICI*_untrained_).

Data were checked for outliers (>3 *SD*), which were removed prior to each analysis. Each variable was tested for normality using the Kolmogorov-Smirnov test, and log transformed (*ln*) in the event of a violation of normality prior to further analysis. The alpha level was set to 0.05 (with a Greenhouse-Geisser degrees of freedom adjustment applied when the assumption of sphericity was violated, i.e., *ε* < 0.7); significant main effects and interactions were explored using *post hoc* pairwise comparisons using the Sidak adjustment. Partial eta-squared and Cohen’s *d* are provided as measures of effect size to aid the interpretation of tests of significance. All data are presented as means and 95% confidence intervals (CI).

## Results

### Performance at Pre-Test and Subsequent Changes in Performance with Training

An initial analysis on *ACC* revealed a significant hand × age interaction, *F*_(1,49)_ = 12.21, *p* = 0.001, ${\text{η}}_{\text{p}}^{\text{2}}$ = 0.199. *Post hoc* comparisons revealed that while for the younger adults acceleration was greater in the trained hand (*M* = 0.34 [0.28, 0.41]) than in the untrained hand (*M* = 0.25 [0.17, 0.33]; *p* = 0.001, *d* = 1.273), there was no such between-hand difference in the older adults (*p* = 0.193). Main effects of hand, feedback and age, and all other interactions were not statistically significant (all *F* < 2.60, all *p* > 0.113).

**Figure 3:**
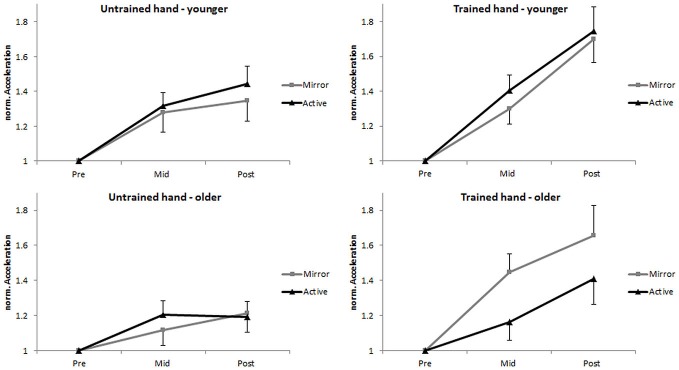
**Normalized (*n*) performance of the untrained (left column) and the trained (right column) hand in the pre-, mid-, and post-test for the young (top row) and the older (bottom row) groups.** Error bars denote SEM.

A subsequent analysis on *nACC* revealed a significant main effect of time, *F*_(1,49)_ = 29.27, *p* < 0.001, ${\text{η}}_{\text{p}}^{\text{2}}$ = 0.374. *Post hoc* comparisons showed that acceleration was greater at post-test (*M* = 1.46 [1.36, 1.57]) when compared to mid-test (*M* = 1.28 [1.20, 1.36]), *p* < 0.001. Furthermore, an interpretation of 95% CI’s indicated that acceleration was greater at mid-test than at pre-test for both the trained hand (*M* = 1.33 [1.23, 1.43]) and the untrained hand (*M* = 1.23 [1.14, 1.32]). A significant main effect of hand revealed greater acceleration in the trained hand (*M* = 1.48 [1.36, 1.60]) compared to the untrained hand (*M* = 1.27 [1.17, 1.36]), *F*_(1,49)_ = 14.96, *p* < 0.001, ${\text{η}}_{\text{p}}^{\text{2}}$ = 0.234. In addition, the time × hand interaction was also found to be significant, *F*_(1,49)_ = 13.33, *p* = 0.001, ${\text{η}}_{\text{p}}^{\text{2}}$ = 0.214. *Post hoc* comparisons revealed that acceleration at mid-test did not differ between the trained hand (*M* = 1.33 [1.23, 1.43]) and the untrained hand (*M* = 1.23 [1.14, 1.32]; *p* = 0.081, *d* = 0.285), while at post-test it was significantly higher in the trained hand (*M* = 1.63 [1.48, 1.78]) than the untrained hand (*M* = 1.30 [1.19, 1.41]; *p* < 0.001, *d* = 0.705). The main effects of age, *F*_(1,49)_ = 2.54, *p* = 0.117, ${\text{η}}_{\text{p}}^{\text{2}}$ = 0.049, and feedback, *F*_(1,49)_ = 1.12, *p* = 0.295, ${\text{η}}_{\text{p}}^{\text{2}}$ = 0.022, were not significant. No other significant interactions were found (all *F* < 2.19, all *p* > 0.146; Figure 3).

### Corticospinal Excitability

**Figure 4:**
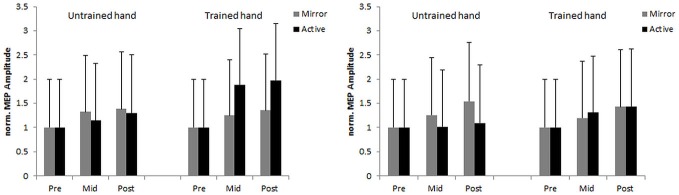
**Normalized and back transformed (*nMEP*) amplitudes evoked in the first dorsal interosseus (FDI) of the trained and the untrained hand for the younger (left side) and the older (right side) groups at pre-, mid-, and post-test.** Error bars denote SEM.

An initial analysis on *MEP* revealed no significant main effects or interactions (all *F* < 1.74, all *p* > 0.194). Since the assumption of normality was violated (on the *nMEP* variable) log transformation was undertaken (i.e., *lnnMEP)* prior to further analysis. The analysis revealed a significant main effect of time, *F*_(1,44)_ = 4.11, *p* = 0.049, ${\text{η}}_{\text{p}}^{\text{2}}$ = 0.084, with greater *lnnMEP* at post-test (*M* = 0.35 [0.24, 0.47]) compared to mid-test (*M* = 0.25 [0.14, 0.37]). In addition, an interpretation of 95% CI’s indicated that *MEP* was greater at mid-test than at pre-test for both the trained hand (*M* = 0.32 [0.16, 0.48]) and the untrained hand (*M* = 0.19 [0.034, 0.35]). Analysis further revealed a trend for hand × feedback interaction, *F*_(1,44)_ = 4.01, *p* = 0.051, ${\text{η}}_{\text{p}}^{\text{2}}$ = 0.082. *Post hoc* comparisons revealed significantly higher *lnnMEP* in the hemisphere responsible for the trained hand (*M* = 0.49 [0.28, 0.69]) compared to the hemisphere responsible for the untrained hand (*M* = 0.13 [−0.09, 0.35]) in the AV condition, *p* = 0.025, *d* = 0.192. No other significant main effects or interactions were found (all *F* < 1.70, all *p* > 0.199; Figure 4).

### Short-Interval Intracortical Inhibition at Pre-Test and Subsequent Changes

An initial analysis on *SICI* revealed a significant feedback × age × hand interaction, *F*_(1,44)_ = 4.49, *p* = 0.041, ${\text{η}}_{\text{p}}^{\text{2}}$ = 0.090. *Post hoc* comparisons revealed significantly lower *SICI* ratio (i.e., greater inhibition) in the trained hemisphere (*M* = 0.56 [0.14, 0.97]) compared to the untrained hemisphere (*M* = 1.13 [0.53, 0.1.73]) in the MV condition in the older adults, *p* = 0.048, *d* = 0.759. No other significant main effect or interactions were found (all *F* < 1.11, all *p* > 0.299).

Subsequent analysis was performed on *lnnSICI* as the assumption of normality on *nSICI* was violated. Data analysis revealed no significant main effects or interactions (all *F* < 2.16, all *p* > 0.149).

### Changes in Performance in the Trained Hand During Training

An analysis performed on *nACCtraining* revealed a significant feedback × age interaction, *F*_(1,49)_ = 8.11, *p* = 0.006, ${\text{η}}_{\text{p}}^{\text{2}}$ = 0.142. *Post hoc* comparisons indicated that in the AV condition significantly smaller performance increases were observed in the older participants (*M* = 1.313 [0.896, 1.730]) than in the younger group (*M* = 2.150 [1.700, 2.600]; *p* = 0.008, *d* = 1.473). The behavioral change for the AV_older_ was also less pronounced when compared to MV_older_ (*M* = 2.068 [1.618, 2.518]; *p* = 0.017, *d* = 0.857).

### Mirror Activation in the Untrained Hand During Training

Based on our exclusion criteria (see “Analysis” Section), one older participant from the MV group was excluded prior to analysis. Subsequent analysis was performed on *lnEMGtraining_untrained_* as the assumption of normality was violated. The level of mirror activity did not differ significantly between the younger and older participants or as a consequence of the provided feedback during training (all *F* < 1.19, all *p* > 0.282).

### Predictors of Performance Change in the Untrained Hand

**Figure 5:**
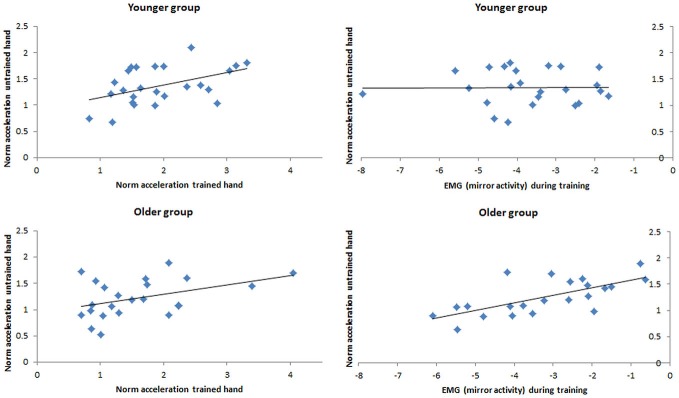
**Simple Correlation (i.e., zero-order Correlation) between the change of performance in the untrained hand at post-test (*ΔACC*_untrained_) and (a) the change of performance in the trained hand during training (*ΔACCtraining*; left side) and (b) the average amount of EMG activity exhibited in the untrained hand (*lnEMGtraining*_untrained_) during training (right side) for the younger group (top row) and the older group (bottom row)**.

Separate multiple regression analyses were employed to identify significant predictors of *ΔACC_untrained_* for the younger and the older adults. For the older adults, analysis revealed that *ΔACC*_untrained_ was significantly predicted by both models (i.e., with and without inclusion of the test-related variables—see “Materials and Methods” Section). The model excluding the test-related variables revealed a better fit and significance (adjusted *R*^2^ = 0.51, *F*_(2,19)_ = 11.81, *p* < 0.001) than the model including all (i.e., test and training) variables (adjusted *R*^2^ = 0.50, *F*_(6, 15)_ = 4.47, *p* = 0.009). In the younger adults *ΔACC*_untrained_ was significantly predicted by the model that included the training-related variables (adjusted *R*^2^ = 0.17, *F*_(2,22)_ = 3.51, *p* = 0.047), but not by the model that was complemented by the test-related variables (*ΔR*^2^ = 0.19, *ΔF*_(4, 18)_ = 1.54, *Δp* = 0.233). In the older adults, *lnEMGtraining*_untrained_, β = 0.604, *t*_(2,19)_ = 3.83, *p* = 0.001, uniquely accounted for a significant portion of the variance in *ΔACC*_untrained_, explaining 36.5% of the variance. In addition, *nACCtraining* was marginally associated with changes in *ΔACC*_untrained_, β = 0.315, *t*_(2,19)_ = 2.00, *p* = 0.061, explaining a further 9.9% of the variance. In the younger adults, only *nACCtraining* accounted for a significant portion of the variance in *ΔACC*_untrained_, β = 0.496, *t*_(2,22)_ = 2.63, *p* = 0.015, explaining 24.6% of the variance (Figure 5).

## Discussion

This study engaged younger and older adults in a ballistic motor training paradigm with their dominant hand and investigated subsequent performance gains (in the same task) in the dominant and the non-dominant hand (i.e., CLT). During the motor learning period participants were provided with different types of visual feedback, either focussing on their active hand, or focussing on a mirror reflection of their active hand, with the aim of facilitating CLT effects.

Participants in both age groups demonstrated an increase in task performance (i.e., peak acceleration) over the duration of the experiment which, in line with previous work (Hinder et al., 2011, 2013; Dickins et al., 2015), was accompanied by a bilateral increase in corticospinal excitability. Further, and consistent with previous findings (for an overview, see Carroll et al., 2006; Lee et al., 2010), the increase in task performance was found to be greater in the trained hand (63% improvement) than in the untrained hand (30% improvement). Moreover, older adults in the current study displayed CLT to an extent that was comparable to the young adults. The current findings therefore suggest that older adults, contrary to previous results (Hinder et al., 2011; Parikh and Cole, 2013), are capable of showing CLT effects to a similar degree to those exhibited by younger adults, a result supported by a recent study by Dickins et al. (2015) that has also demonstrated preserved transfer for older adults in both a complex and a simple motor task.

Unlike the current experiment, where we specifically asked participants to either continuously focus on their active hand or on a mirror image of their active hand during task performance, neither of the two previous studies (Hinder et al., 2011; Parikh and Cole, 2013) provided explicit instructions with regard to the focus of attention during the training. However, the provision of feedback about task performance on a computer screen in those studies suggests that at least some focus was directed away from the hands. As older adults have been shown to be more reliant on visual feedback for accurate motor performance (Swinnen et al., 1998; Voelcker-Rehage, 2008), it is possible that for our older adults focussing on the active hand or a mirror image of the active hand (rather than focussing on a computer screen) represented a beneficial factor leading to similar performance gains in the trained and untrained hand as in the younger adults. Indeed, in younger participants, prior observation of a motor action has been shown to be beneficial for subsequent motor learning in the absence of movement execution (Mattar and Gribble, 2005; Stefan et al., 2005). With regard to ageing, Celnik et al. (2006) recently demonstrated that combining action observation and motor training augmented those training effects obtained by motor training alone, conceivably through a strengthened input to M1 from ventral premotor cortex (through action observation) and supplementary motor area and dorsal premotor cortex (through action execution). It is conceivable, therefore that for the older adults in previous studies (Hinder et al., 2011; Parikh and Cole, 2013), not focusing continually on the hands—while not appearing to decrease performance gains in the *trained* hand—affected the mechanisms of learning and thus, affected (precluded) subsequent transfer. We acknowledge the possibility that our group sizes, although common in TMS studies (e.g., Hinder et al., 2011, 2013; Parikh and Cole, 2013; Dickins et al., 2015), may have contributed to the absence of statistically significant effects of age. Nonetheless, we believe that a continuous focus on the executing hand (as applied in the current study) might have facilitated our participants to internalize crucial movement parameters more effectively, subsequently enabling them to also show performance improvements in the *untrained* hand.

For the current task the provision of augmented visual feedback via MVF, rather than “standard” visual feedback, did not significantly enhance CLT in our younger or older participants. It is possible that our ballistic finger movement task did not elicit enhanced performance improvements in the untrained hand in the MVF condition because online visual feedback was neither a prerequisite for accurate completion of the task, nor was it necessary to drive performance improvements. It is conceivable that the provision of visual feedback was helpful during the very early stages of the training period, in which participants acquired the basic “structure” of the simple movement task, but the feedback did not contribute to subsequent performance improvements. MVF may promote enhanced learning and facilitate CLT if used in conjunction with a more demanding task (perhaps requiring online modifications and feedback control) in which visual feedback has been shown to be most beneficial (for an overview, see Sigrist et al., 2013). Consistent with this supposition, in recent studies showing beneficial effects of MVF on CLT, participants were engaged in motor tasks requiring and/or profiting from online visual feedback, such as moving marbles with a spoon, putting elastic bands over a glass, or rotating two balls in one hand as quickly as possible (Lappchen et al., 2012; Nojima et al., 2012). Transfer in tasks involving forceful contractions, such as the ballistic task employed here, or strength training protocols (e.g., Farthing et al., 2007) may be less influenced by manipulations in visual feedback.

While the lack of a significant effect of visual feedback in mediating the extent of CLT may have been caused by a lack of task complexity, it is also possible that other factors may be more important in facilitating CLT across the lifespan. In the older adults the extent of bilateral activation (mirror activity) during the training period significantly accounted for subsequent performance gains in the untrained hand (accounted for 36.5% of the variance), while the extent of performance improvements in the trained hand was weakly associated with the subsequent transfer. The current results thus suggest that, for older adults, unintended activation of the ipsilateral hand during unilateral training appears to be crucial in increasing subsequent motor performance in the untrained hand. Greater bilateral muscle activity during unilateral tasks in the older adults is well known and has previously been shown for a variety of different movement tasks (Mattay et al., 2002; Bodwell et al., 2003; Ward and Frackowiak, 2003; Hoy et al., 2004; Baliz et al., 2005; Hinder et al., 2011). This increase in motor overflow is most likely caused by neurological changes in the healthy ageing brain, such as a reduced integrity of the corpus callosum (Hoy et al., 2004) resulting in bilateral cortical activity. Overactivation of (bilateral) brain areas not primarily involved for task execution has previously been shown to be associated with better task performance in the elderly in studies employing simple (Mattay et al., 2002; Ward and Frackowiak, 2003) and complex motor tasks (Bodwell et al., 2003). In line with those experiments, the current study suggests that bilateral muscle activation is also important for the *transfer* of simple motor tasks in older adults (Figure 5). In accordance with the HAROLD model (Cabeza, 2002), which describes less lateralized prefrontal activation to be associated with increased cognitive task performance in older adults, we propose that the exhibited bilateral activation can be considered a compensatory mechanism to ensure bilateral performance improvements after unilateral movement tasks. Despite the fact that the task under investigation (suggested to be predominantly M1-driven) did not result in age-dependent changes in M1, according to the above mentioned studies we assume that an unconscious bilateral cortical activation in other brain areas (not assessed here) that project directly or indirectly onto M1 may have promoted bilateral muscle activity in the older adults and ensured performance improvements in both the trained and the untrained hand. Support for this hypothesis comes from previous studies that (additionally to prefrontal areas) have shown greater bilateral activation in sensorimotor cortex during unilateral motor tasks to be beneficial for motor performance in older adults (Mattay et al., 2002; Naccarato et al., 2006). Our proposal of bilateral activation being a compensatory rather than a mal-adaptive mechanism for CLT in the older adults is also in accordance with a study by Graziadio et al. (2015). In their study the authors found *similar* transfer in young and older groups in movement tasks that have been shown to result in bilateral cortical activation in both age groups, but *increased* transfer in the older compared to the younger adults for movement tasks that are known to cause unilateral activation in the younger and bilateral activation in the older adults. Based on those results it was suggested that the age-related bilateral activation involved the recruitment of neural circuits available to both hands and therefore facilitated subsequence transfer of the learned motor skill from the trained to the untrained hand. The current findings support and extend those obtained by Graziadio et al. (2015) through neurophysiological results.

In the current study, whereas older adults’ performance gains in the untrained hand seem dependent, at least to some degree, on prior unintended muscular activation of that hand, CLT does not appear contingent upon bilateral activation in the younger adults. This latter result is in accord with a recent review that has suggested bilateral activation as a non-essential process for CLT in young people (Zult et al., 2014). Rather, and consistent with previous research (Lee et al., 2010; Hinder et al., 2011, 2012), in the current study younger adults’ performance increases in the untrained hand were found to be contingent upon (i.e., correlated to) training related gains in the trained hand (accounted for 24.6% of the variance). That is, younger participants showing the greatest performance gains during training subsequently demonstrated higher increases in motor performance in the untrained hand.

In light of the current results, changes in the nature of the neural mechanisms mediating CLT may occur as part of the healthy ageing process. With regard to potential mechanisms of CLT, current hypotheses either suggest changes in the untrained hemisphere (i.e., cross-activation hypothesis) or changes in the trained hemisphere, accessible by the untrained hemisphere (i.e., bilateral access hypothesis) as a requirement for successful transfer (for more detail, see Lee et al., 2010). Our results demonstrate bilateral increases in corticospinal excitability following unilateral practice across all groups; however, these increases did not predict the extent of CLT. This finding is in accordance with a previous study by Dickins et al. (2015), who also only found a marginally-reliable relationship between changes in corticospinal excitability in the untrained hemisphere and CLT in the younger and no such association in the older adults. Rather, a strong relationship between mirror activation and performance changes in the untrained hand in the older participants was observed. Accordingly, it may be the case that bilateral muscle activity recorded *during* a task is a more sensitive marker of bilateral activation than measures of corticospinal excitability determined at rest following training, which may be influenced by numerous other factors. While increases in corticospinal excitability in the untrained hemisphere were causally related to transfer in younger adults in a previous study (Lee et al., 2010), the fact we did not find a similar relationship indicates that the relationship between performance increases and excitability may be quite variable.

The finding of a significant relationship between mirror activity and CLT is consistent with the cross-activation theory (Carroll et al., 2008; Lee et al., 2010; Hinder et al., 2011), whereby unilateral-training-induced activations in the trained and untrained hemispheres specifically mediate the contralateral (i.e., trained or untrained) limb’s performance, respectively (Lee et al., 2010). That is, activation of the untrained hemisphere are likely to drive successful CLT and subsequent performance improvements in the untrained hand (Carroll et al., 2008; Lee et al., 2010; Hinder et al., 2011; Wiestler et al., 2014). Interestingly, our findings demonstrating an association between bilateral muscle activation and CLT in the older adults appear contradictory to the results obtained by an earlier study (Hinder et al., 2011) at first view, which did not find any such relationship. However, while bilateral EMG activity in the current study was recorded during the training period, in the experiment conducted by Hinder et al. (2011), it was collected during the test-period. The possibility therefore exists that, in line with the cross-activation hypothesis, processes (i.e., bilateral muscle activation) occurring *during* the actual intervention period, but not *after* training, might drive cross-limb adaptations and thus underpin subsequent performance gains. In the current study, despite the fact that young and older adults exhibited comparable levels of performance increase in the trained hand and similar levels of mirror activity in the untrained (inactive) limb, the finding that these parameters relate differently to subsequent CLT is suggestive of subtle changes in the factors mediating CLT. Moreover, possible changes in the balance of the mechanisms underlying transfer, together with shifts in the level of action of these mechanisms (e.g., cortical, corticospinal or spinal) may occur as a function of healthy ageing.

Considering the purported changes in the mechanisms underlying CLT that occur across the lifespan, future research is warranted comparing the neuronal activation of regions, such as M1 or dorsal premotor cortex, which are presumably activated during the learning and retrieval of a unilateral movement task and the subsequent performance in the untrained hand in younger and older adults.

Based on the current finding that bilateral (muscle) activation appears to be a driving factor in eliciting CLT in the older (but not the younger) adults, future work could also aim to manipulate the amount of mirror movements during a unilateral ballistic movement task and assess the corresponding change in transfer. Non-invasive brain stimulation techniques could be used to up- or down-regulate presumably active brain regions in order to elucidate causal relationships between CLT and mirror activation. Such findings would improve our understanding of the neural underpinnings of CLT and subsequently enhance clinical applicability.

Finally, to further investigate the effects of augmented visual feedback (i.e., MVF) on CLT, future work could focus on a variety of more complex tasks requiring online modification of motor commands on the basis of visual feedback. Having demonstrated that focusing attention on either the active hand or a mirror image of the active hand *can* evoke transfer in older adults, such an approach would enable us to determine whether further enhancement of transfer by way of MVF is possible for certain tasks that mimic complex everyday movements (e.g., reaching and grasping), which are vital to maintain independent living in later life, but often affected severely following brain injury (e.g., stroke) or following falls and subsequent limb immobilization.

## Conflict of Interest Statement

The authors declare that the research was conducted in the absence of any commercial or financial relationships that could be construed as a potential conflict of interest.

## References

[B1] Altschuler E. L. Wisdom S. B. Stone L. Foster C. Galasko D. Llewellyn D. M. E. (1999). Rehabilitation of hemiparesis after stroke with a mirror. Lancet 353, 2035–2036. 10.1016/s0140-6736(99)00920-4 10376620

[B2] Baliz Y. Armatas C. Farrow M. Hoy K. E. Fitzgerald P. B. Bradshaw J. L. (2005). The influence of attention and age on the occurrence of mirror movements. J. Int. Neuropsychol. Soc. 11, 855–862. 10.1017/s1355617705051003 16519264

[B3] Bodwell J. A. Mahurin R. K. Waddle S. Price R. Cramer S. C. (2003). Age and features of movement influence motor overflow. J. Am. Geriatr. Soc. 51, 1735–1739. 10.1046/j.1532-5415.2003.51557.x 14687351

[B4] Cabeza R. (2002). Hemispheric asymmetry reduction in older adults: the HAROLD model. Psychol. Aging 17, 85–100. 10.1037/0882-7974.17.1.85 11931290

[B5] Carroll T. J. Barry B. Riek S. Carson R. G. (2001). Resistance training enhances the stability of sensorimotor coordination. Proc. Biol. Sci. 268, 221–227. 10.1098/rspb.2000.1356 11217890 PMC1088595

[B6] Carroll T. J. Herbert R. D. Munn J. Lee M. Gandevia S. C. (2006). Contralateral effects of unilateral strength training: evidence and possible mechanisms. J. Appl. Physiol. (1985) 101, 1514–1522. 10.1152/japplphysiol.00531.2006 17043329

[B7] Carroll T. J. Lee M. Hsu M. Sayde J. (2008). Unilateral practice of a ballistic movement causes bilateral increases in performance and corticospinal excitability. J. Appl. Physiol. (1985) 104, 1656–1664. 10.1152/japplphysiol.01351.2007 18403447

[B8] Carson R. G. Ruddy K. L. (2012). Vision modulates corticospinal suppression in a functionally specific manner during movement of the opposite limb. J. Neurosci. 32, 646–652. 10.1523/JNEUROSCI.4435-11.2012 22238100 PMC6621083

[B9] Celnik P. Stefan K. Hummel F. Duque J. Classen J. Cohen L. G. (2006). Encoding a motor memory in the older adult by action observation. Neuroimage 29, 677–684. 10.1016/j.neuroimage.2005.07.039 16125417

[B10] Dettmers C. Ridding M. C. Stephan K. M. Lemon R. N. Rothwell J. C. Frackowiak R. S. J. (1996). Comparison of regional cerebral blood flow with transcranial magnetic stimulation at different forces. J. Appl. Physiol. (1985) 81, 596–603. 8872623 10.1152/jappl.1996.81.2.596

[B11] Dickins D. S. E. Sale M. V. Kamke M. R. (2015). Intermanual transfer and bilateral cortical plasticity is maintained in older adults after skilled motor training with simple and complex tasks. Front. Aging Neurosci. 7:73. 10.3389/fnagi.2015.00073 25999856 PMC4423452

[B12] Farthing J. P. (2009). Cross-education of strength depends on limb dominance: implications for theory and application. Exerc. Sport Sci. Rev. 37, 179–187. 10.1097/JES.0b013e3181b7e882 19955867

[B13] Farthing J. P. Borowsky R. Chilibeck P. D. Binsted G. Sarty G. E. (2007). Neuro-physiological adaptations associated with cross-education of strength. Brain Topogr. 20, 77–88. 10.1007/s10548-007-0033-2 17932739

[B14] Fujiyama H. Garry M. I. Levin O. Swinnen S. P. Summers J. J. (2009). Age-related differences in inhibitory processes during interlimb coordination. Brain Res. 1262, 38–47. 10.1016/j.brainres.2009.01.023 19368842

[B15] Garry M. I. Thomson R. H. S. (2009). The effect of test TMS intensity on short-interval intracortical inhibition in different excitability states. Exp. Brain Res. 193, 267–274. 10.1007/s00221-008-1620-5 18974984

[B16] Garry M. I. Loftus A. Summers J. J. (2005). Mirror, mirror on the wall: viewing a mirror reflection of unilateral hand movements facilitates ipsilateral M1 excitability. Exp. Brain Res. 163, 118–122. 10.1007/s00221-005-2226-9 15754176

[B17] Graziadio S. Nazarpour K. Gretenkord S. Jackson A. Eyre J. A. (2015). Greater intermanual transfer in the elderly suggests age-related bilateral motor cortex activation is compensatory. J. Mot. Behav. 47, 47–55. 10.1080/00222895.2014.981501 25575222 PMC4299868

[B170] Hinder M. R. Carroll T. J. Summers J. J. (2013). Inter-limb transfer of ballistic motor skill following non-dominant limb training in young and older adults. Exp. Brain Res. 227, 19–29. 10.1007/s00221-013-3481-9 23535836

[B18] Hinder M. R. Fujiyama H. Summers J. J. (2012). Premotor-motor interhemispheric inhibition is released during movement initiation in older but not young adults. Plos One 7:e52573. 10.1371/journal.pone.0052573 23285097 PMC3526571

[B19] Hinder M. R. Schmidt M. W. Garry M. I. Carroll T. J. Summers J. J. (2011). Absence of cross-limb transfer of performance gains following ballistic motor practice in older adults. J. Appl. Physiol. 110, 166–175. 10.1152/japplphysiol.00958.2010 21088207

[B20] Howatson G. Zult T. Farthing J. P. Zijdewind I. Hortobágyi T. (2013). Mirror training to augment cross-education during resistance training: a hypothesis. Front. Hum. Neurosci. 7:396. 10.3389/fnhum.2013.00396 23898251 PMC3721498

[B21] Hoy K. E. Fitzgerald P. B. Bradshaw J. L. Armatas C. A. Georgiou-Karistianis N. (2004). Investigating the cortical origins of motor overflow. Brain Res. Brain Res. Rev. 46, 315–327. 10.1016/j.brainresrev.2004.07.013 15571773

[B210] Kujirai T. Caramia M. D. Rothwell J. C. Day B. L. Thompson P. D. Ferbert A. (1993). Corticocortical inhibition in human motor cortex. J. Physiol. 471, 501–519. 10.1113/jphysiol.1993.sp019912 8120818 PMC1143973

[B22] Lappchen C. H. Ringer T. Blessin J. Seidel G. Grieshammer S. Lange R. (2012). Optical illusion alters M1 excitability after mirror therapy: a TMS study. J. Neurophysiol. 108, 2857–2861. 10.1152/jn.00321.2012 22972955

[B23] Lee M. Hinder M. R. Gandevia S. C. Carroll T. J. (2010). The ipsilateral motor cortex contributes to cross-limb transfer of performance gains after ballistic motor practice. J. Physiol. 588, 201–212. 10.1113/jphysiol.2009.183855 19917563 PMC2821559

[B24] Mattar A. A. G. Gribble P. L. (2005). Motor learning by observing. Neuron 46, 153–160. 10.1016/j.neuron.2005.02.009 15820701

[B25] Mattay V. S. Fera F. Tessitore A. Hariri A. R. Das S. Callicott J. H. (2002). Neurophysiological correlates of age-related changes in human motor function. Neurology 58, 630–635. 10.1212/wnl.58.4.630 11865144

[B26] McCabe C. S. Haigh R. C. Ring E. F. J. Halligan P. W. Wall P. D. Blake D. R. (2003). A controlled pilot study of the utility of mirror visual feedback in the treatment of complex regional pain syndrome (type 1). Rheumatology (Oxford) 42, 97–101. 10.1093/rheumatology/keg041 12509620

[B27] Muellbacher W. Facchini S. Boroojerdi B. Hallett M. (2000). Changes in motor cortex excitability during ipsilateral hand muscle activation in humans. Clin. Neurophysiol. 111, 344–349. 10.1016/s1388-2457(99)00243-6 10680571

[B28] Naccarato M. Calautti C. Jones P. S. Day D. J. Carpenter T. A. Baron J. C. (2006). Does healthy aging affect the hemispheric activation balance during paced index-to-thumb opposition task? An fMRI study. Neuroimage 32, 1250–1256. 10.1016/j.neuroimage.2006.05.003 16806984

[B29] Nojima I. Mima T. Koganemaru S. Thabit M. N. Fukuyama H. Kawamata T. (2012). Human motor plasticity induced by mirror visual feedback. J. Neurosci. 32, 1293–1300. 10.1523/JNEUROSCI.5364-11.2012 22279214 PMC6796271

[B30] Parikh P. J. Cole K. J. (2013). Transfer of learning between hands to handle a novel object in old age. Exp. Brain Res. 227, 9–18. 10.1007/s00221-013-3451-2 23595702

[B31] Ramachandran V. S. Altschuler E. L. (2009). The use of visual feedback, in particular mirror visual feedback, in restoring brain function. Brain 132, 1693–1710. 10.1093/brain/awp135 19506071

[B310] Ramachandran V. S. Rogers-Ramachandran D. (1996). Synaesthesia in phantom limbs induced with mirrors. Pro. Biol. Sci. 263, 377–386. 10.1098/rspb.1996.0058 8637922

[B32] Reissig P. Garry M. I. Summers J. J. Hinder M. R. (2014). Visual feedback-related changes in ipsilateral cortical excitability during unimanual movement: implications for mirror therapy. Neuropsychol. Rehabil. 24, 936–957. 10.1080/09602011.2014.922889 24894429

[B33] Ruddy K. L. Carson R. G. (2013). Neural pathways mediating cross education of motor function. Front. Hum. Neurosci. 7:397. 10.3389/fnhum.2013.00397 23908616 PMC3725409

[B34] Schmidt R. A. Lee T. D. (2011). Motor Control and Learning: A Behavioual Emphasis. Illinois: Human Kinetics

[B35] Seidler R. D. Bernard J. A. Burutolu T. B. Fling B. W. Gordon M. T. Gwin J. T. (2010). Motor control and aging: links to age-related brain structural, functional and biochemical effects. Neurosci. Biobehav. Rev. 34, 721–733. 10.1016/j.neubiorev.2009.10.005 19850077 PMC2838968

[B36] Selvanayagam V. S. Riek S. Carroll T. J. (2011). Early neural responses to strength training. J. Appl. Physiol. (1985) 111, 367–375. 10.1152/japplphysiol.00064.2011 21551014

[B37] Sigrist R. Rauter G. Riener R. Wolf P. (2013). Augmented visual, auditory, haptic and multimodal feedback in motor learning: a review. Psychon. Bull. Rev. 20, 21–53. 10.3758/s13423-012-0333-8 23132605

[B38] Stefan K. Cohen L. G. Duque J. Mazzocchio R. Celnik P. Sawaki L. (2005). Formation of a motor memory by action observation. J. Neurosci. 25, 9339–9346. 10.1523/jneurosci.2282-05.2005 16221842 PMC6725701

[B39] Swinnen S. P. Verschueren S. M. P. Bogaerts H. Dounskaia N. Lee T. D. Stelmach G. E. (1998). Age-related deficits in motor learning and differences in feedback processing during the production of a bimanual coordination pattern. Cogn. Neuropsychol. 15, 439–466. 10.1080/026432998381104 28657466

[B40] Talelli P. Waddingham W. Ewas A. Rothwell J. C. Ward N. S. (2008). The effect of age on task-related modulation of interhemispheric balance. Exp. Brain Res. 186, 59–66. 10.1007/s00221-007-1205-8 18040671 PMC2257995

[B41] Voelcker-Rehage C. (2008). Motor-skill learning in older adults - a review of studies on age-related differences. Eur. Rev. Aging Phys. Act. 5, 5–16. 10.1007/s11556-008-0030-9

[B42] Ward N. S. Frackowiak R. S. J. (2003). Age-related changes in the neural correlates of motor performance. Brain 126, 873–888. 10.1093/brain/awg071 12615645 PMC3717766

[B43] Wiestler T. Waters-Metenier S. Diedrichsen J. (2014). Effector-independent motor sequence representations exist in extrinsic and intrinsic reference frames. J. Neurosci. 34, 5054–5064. 10.1523/JNEUROSCI.5363-13.2014 24695723 PMC3972728

[B44] Yavuzer G. Selles R. Sezer N. Sütbeyaz S. Bussmann J. B. Köseoğlu F. (2008). Mirror therapy improves hand function in subacute stroke: a randomized controlled trial. Arch. Phys. Med. Rehabil. 89, 393–398. 10.1016/j.apmr.2007.08.162 18295613

[B45] Zult T. Howatson G. Kádár E. E. Farthing J. P. Hortobágyi T. (2014). Role of the mirror-neuron system in cross-education. Sports Med. 44, 159–178. 10.1007/s40279-013-0105-2 24122078

